# Mortality Rate in Breast Implant Surgery: Is an Additional Procedure Worthwhile to Mitigate BIA-ALCL Risk?

**DOI:** 10.1007/s00266-022-03138-5

**Published:** 2022-11-14

**Authors:** Fabio Santanelli di Pompeo, Michail Sorotos, Mark W. Clemens, Guido Paolini, Paolo Anibaldi, Marina Davoli, Giovanni Baglio, Luigi Pinnarelli, Margherita Ferranti, Francesco Cerza, Stefano Domenico Cicala, Guido Firmani

**Affiliations:** 1 Faculty of Medicine and Psychology, Neurosciences, Mental Health and Sensory Organ (NESMOS) Department, Sapienza University of Rome, Rome, Italy; 2 Sant’Andrea University Hospital, Rome, Italy; 3 The University of Texas MD Anderson Cancer Center, Houston, Texas USA; 4 Lazio Regional Health Service, Rome, Italy; 5 Italian National Agency for Regional Healthcare Services (AGENAS), Italian Ministry of Health, Rome, Italy

**Keywords:** Breast Implant Surgery, Mortality, Macrotextured, Breast Explantation, BIA-ALCL

## Abstract

**Background:**

Because of poor knowledge of risks and benefits, prophylactic explantation of high BIA-ALCL risk breast implant (BI) is not indicated. Several surgical risks have been associated with BI surgery, with mortality being the most frightening. Primary aim of this study is to assess mortality rate in patients undergoing breast implant surgery for aesthetic or reconstructive indication.

**Materials and Methods:**

In this retrospective observational cohort study, Breast Implant Surgery Mortality rate (BISM) was calculated as the perioperative mortality rate among 99,690 patients who underwent BI surgery for oncologic and non-oncologic indications. Mean age at first implant placement (A1P), implant lifespan (IL), and women’s life expectancy (WLE) were obtained from a literature review and population database.

**Results:**

BISM rate was 0, and mean A1P was 34 years for breast augmentation, and 50 years for breast reconstruction. Regardless of indication, overall mean A1P can be presumed to be 39 years, while mean BIL was estimated as 9 years and WLE as 85 years.

**Conclusion:**

This study first showed that the BISM risk is 0. This information, and the knowledge that BI patients will undergo one or more revisional procedures if not explantation during their lifetime, may help surgeons in the decision-making process of a pre-emptive substitution or explant in patients at high risk of BIA-ALCL. Our recommendation is that patients with existing macrotextured implants do have a relative indication for explantation and total capsulectomy. The final decision should be shared between patient and surgeon following an evaluation of benefits, surgical risks and comorbidities.

**Level of Evidence IV:**

This journal requires that authors assign a level of evidence to each article. For a full description of these Evidence-Based Medicine ratings, please refer to the Table of Contents or the online Instructions to Authors www.springer.com/00266*.*

## Introduction

The World Consensus Conferences on Breast Implant–Associated Anaplastic Large Cell Lymphoma (BIA-ALCL), held in 2019, 2020 and 2021, aimed to raise disease awareness and to create a forum for research on treatment and pathogenesis. As of May 2021, data from the American Society of Plastic Surgery (ASPS) BIA-ALCL Global Network and the European Association of Plastic Surgeons Scientific Committee on Device Safety and Development, report 1,148 confirmed cases worldwide, of which 489 presented in Europe [1]. The Scientific Committee on Health, Emerging and Environmental Risks (SCHEER) of the European Commission on 2021 issued a final opinion claiming a causal relationship with a moderate weight of evidence between all textured breast implants (BIs) and BIA-ALCL, and a higher risk in individuals with intermediate-to-high surface roughness implants (according to ISO-14607:2018) [2]. This statement did not address prophylactic explantation of asymptomatic patients with textured implants for risk reduction, an increasingly controversial practice.

For most textured implant manufacturers, specific device risk is unknown with the exception of Biocell textured implants which appear to have a risk of BIA-ALCL of 1 in 350 [3]. Many plastic surgeons and patients have pursued an explantation strategy for risk reduction, with prophylactic removal/replacement and variable removal of the surrounding scar capsule [4]. Is an additional BI procedure worthwhile to mitigate the risk of BIA-ALCL, or do the surgical risks outweigh the perceived benefits? Regulatory authorities often highlighted the importance of surgical risks in contrast to the rare incidence of neoplastic occurrence, leaving the decision to patients and physicians [5].

Several surgical risks have been addressed regarding implant-based breast surgery, such as pneumothorax, deep vein thrombosis, infection, breast pain. Perioperative mortality is the most concerning [6]. Mortality risk has not been reported and only extrapolated for aesthetic surgery or for cosmetic breast surgery procedures [7], but not specifically for BI surgeries.

The primary aim of this study was to specifically assess *perioperative mortality* in patients who underwent aesthetic or reconstructive primary or revisional BI surgeries. Secondary aim was to determine the mean age at first BI placement (A1P), breast implant lifespan (BIL), and the overall women life expectancy (WLE) by literature review. An understanding of the mortality rate of revisionary breast implant surgery would provide patients with data necessary to decide on the risks and benefits of prophylactic explantation. This knowledge may further assist surgeons discussing prophylactic explantation in patients with high-risk BIs in accordance with recommendations from SCHEER.

## Materials and Methods

### Breast Implant-Related Mortality Study

This retrospective observational cohort study was conducted in accordance with the World Medical Association’s Declaration of Helsinki. *Perioperative mortality* was defined as the all-cause death rate prior to discharge among patients who underwent one or more procedures in an operating theater during the relevant admission. Our primary endpoint, BI Surgery Mortality (BISM) rate, was defined accordingly as the rate of perioperative deaths during the relevant admission, in patients undergoing any procedures using BI. Perioperative deaths for unrelated causes to BI surgery were excluded.

All BI procedures performed in Italy between January 1, 2012, and December 31, 2019, were included. Information was anonymized, according to the European Union’s General Data Protection Regulation n. 2016/679. Clinical and demographic data from public and private hospitals in all 20 regions of Italy were extracted from Hospital Information Systems, Sanitary Emergency Information Systems, Specialist Assistance Information Systems, and Tax Registries. The perioperative mortality was obtained by analyzing the hospital discharge form of each patient.

Patients were divided into 2 cohorts of BI surgery: (A) oncologic and (B) non-oncologic. Codes to assign patients to these cohorts were searched from the International Classification of Diseases, 9th Revision, Clinical Modifications (ICD-9-CM), currently used in Italy, and are listed in Table 1. When only neutral codes were found, we searched for secondary procedure codes among the above-mentioned ones to guide cohort assignment. If none were present, we used a contingency strategy of searching for specific diagnostic codes to guide cohort assignment. Patients whose records did not include any of the above-mentioned ICD-9-CM procedure codes were excluded (Table 2).

**Table 1:** International Classification of Diseases, 9th Revision, Clinical Modifications (ICD-9-CM) procedure codes used for allocation of patients in cohort groups for national-level study.

ICD-9-CM CODE	Description of the procedure code
*Cohort A*^a^: *Cancer patients*	
85.33	Unilateral subcutaneous mammectomy with synchronous implant convert
85.35	Bilateral subcutaneous mammectomy with synchronous implant
85.95	Insertion of breast tissue expander
85.96	Removal of breast tissue expander
*Cohort B*^b^: *Non-cancer patients*	
85.50	Augmentation mammoplasty
*Neutral codes*^c^: —*not sufficient for allocation in cohort groups*	
85.93	Revision of implant of breast
85.94	Removal of implant of breast
85.53	Unilateral breast implant
85.54	Bilateral breast implant

^a^**Cohort A** includes patients who underwent breast implant placement, revision or removal for reconstruction following breast cancer

^b^**Cohort B** includes breast implant placement, revision or removal for non-oncologic purposes (i.e., cosmetic breast augmentations, correction of congenital breast deformities or other purposes)

^c^**Neutral codes** are deemed sufficient for recruitment in the study, but insufficient for allocation to a specific cohort group

**Table 2:** International Classification of Diseases, 9th Revision, Clinical Modifications (ICD-9-CM) diagnostic codes used for allocation of patients in cohort groups for national-level study.

ICD-9-CM CODE	Description of the diagnosis code
*Cohort A*^a^: *cancer patients*	
233.0	Cancer in situ of breast
239.3	Neoplasm of unspecified nature: breast.
174.0	Malignant neoplasm of nipple and areola of female breast
174.1	Malignant neoplasm of central portion of female breast
174.2	Malignant neoplasm of upper-inner quadrant of female breast
174.3	Malignant neoplasm of lower-inner quadrant of female breast
174.4	Malignant neoplasm of upper-outer quadrant of female breast
174.5	Malignant neoplasm of lower-outer quadrant of female breast
174.6	Malignant neoplasm of axillary tail of female breast
174.8	Malignant neoplasm of other specified sites of female breast
174.9	Malignant neoplasm of breast (female), unspecified
612/0	Deformity and disproportion of reconstructed breast
V10.3	Personal history of malignant neoplasm of breast
v43.82	Breast replacement
V45.71	Acquired absence of breast and nipple
V84.01	Genetic susceptibility to malignant neoplasm of breast
*Cohort B*^b^: *Non-cancer patients*	
757.6	Specified congenital anomalies of breast
611.82	Hypoplasia of breast
611.89	Other specified disorders of breast
756.81	Absence of muscle and tendon [used for Poland syndrome]
*Neutral codes*^c^—*not sufficient for allocation in cohort groups*	
V51	Aftercare involving the use of plastic surgery
v52.4	Fitting and adjustment of breast prosthesis and implant

^a^**Cohort A** includes patients who underwent breast implant placement, revision or removal for reconstruction following breast cancer

^b^**Cohort B** includes breast implant placement, revision or removal for non-oncologic purposes

^c^**Neutral codes** are deemed sufficient for recruitment in the study, but insufficient for allocation to a specific cohort group

Descriptive statistical analyses were performed using StatView (version 5.0) and SAS (version 9.4) software (both from SAS Institute, Inc., Cary, NC, USA).

### Review of Literature

We performed a literature review using the PubMed, Embase, Web of Science (including Science Citation Index and Conference Proceedings Citation Index), and SCOPUS (Health Sciences and Physical Sciences) databases to identify publications regarding BI epidemiology. The search was conducted from May to October 2021, with no limitations on the publication date. Search strategies with inclusion and exclusion criteria are described in Table 3. Only articles on humans and written in English were considered. WLE was obtained through publicly available statistics [8]. The search was conducted by G.F., G.F. and M.S., who independently reviewed the titles and abstracts, and then selected articles based on inclusion and exclusion criteria. Disagreements were resolved through consensus-based discussion with a third reviewer, F.S.d.P.

**Table 3:** Search strategies for review of literature, subdivided according to academic search engine.

Academic search engine	Specific search strategies
PubMed	((Breast implant*[tiab]) AND (surgery[MeSH]) AND (epidemiology[MeSH] OR silicone gels/adverse effects[MeSH] OR silicones/adverse effects[MeSH])) OR ((breast AND (implant or implants or prostheses* or endoprosthes*)) AND (epidemiology* or prevalence* or incidence* or risk*)
Embase	(((breast AND endoprosthesis/exp) OR (breast AND implant*) OR (breast* AND silicon*)) AND (epidemiology*))
Web of Science	Topic=(breast AND epidemiology*) AND Topic=(implant* OR prosthes* OR endoprosthes* OR silicon*) AND Topic=(epidemiology* or prevalence* or incidence* or risk* or *mortality)
SCOPUS	(TITLE-ABS-KEY(breast AND epidemiology*) AND TITLE-ABS-KEY((implant* OR prosthes* OR endoprosthes* OR silicon*)) AND TITLE-ABS-KEY((epidemiology* or prevalence* or incidence* or risk*)))

## Results

### Breast Implant-Related Mortality Study

A total of 99,690 procedures were included, of which 57,369 assigned to cohort A (oncologic) and 42,321 to cohort B (non-oncologic). One case of perioperative mortality was identified in cohort A, but upon review of anonymous clinical data and hospital discharge forms, mortality was attributed to chest wall infiltration of a radiation-induced angiosarcoma, and therefore excluded. No cases were recorded in cohort B, leading to an overall mortality rate of 0:99,690 surgeries.

### Review of Literature for Breast Implant Epidemiology

Of 1,277 potentially relevant manuscripts, 894 were excluded because not relevant to the topic and 383 included, but only 54 presented pertinent epidemiologic data.

### Age at First Breast Implant Placement (A1P)

According to ASPS (2018), the mean A1P in patients who underwent cosmetic breast augmentation in the USA is 34 years [9]. According to studies, the age for breast reconstruction ranges between 48 and 52 years (mean 50 years) [10, 11]. Since the BIs augmentation-to-reconstruction ratio in the USA is 75% versus 25% [6], and in Italy 63% versus 37%, the mean A1P, regardless of indication, can be estimated as 34 × 0.75 + 50 × 0.25 = 38 years in USA, and 34 × 0.63 + 50 × 0.37 = 40 years in Italy.

### Breast Implant Lifespan (BIL)

BIs are not lifetime devices, and the longer the indwelling, the likelier patients will experience adverse outcomes requiring revision. In 1998, Goodman et al [12] analyzed 1099 implants and found that BIL was 16.4 years. In 2000, Benadiba et al. [13] retrospectively analyzed 949 implants and reported a BIL of 10.6 years. More recent long-term data can be found in large post-approval and manufacturer-funded core studies, including Caplin et al.[14], who followed 1008 patients (1898 implants) for 10 years and estimated a BIL of 7.9–8.3 years. By averaging the two largest reports on the latest generation of implants [13, 14], BIL, defined as the period time between insertion and rupture or removal, was estimated at 9.34 years.

### Women’s Life Expectancy

Finally, WLE was found to be about 84.5 years in the USA and 85.4 years in Italy [8].

## Discussion

Our study is the first to define that cosmetic or reconstructive BI surgeries have a perioperative mortality rate of effectively zero, and are therefore relatively safe and in line with post-market approval data. Recently, the SCHEER demonstrated a causal relationship between BIA-ALCL and textured surface breast implants, characterizing the weight of evidence as *moderate* and “sufficient” based upon data from a primary line of evidence, but acknowledged gaps from a lack of prospective randomized controlled trials falling short of a *strong* weight [2]. Primary line was based on epidemiological studies, mostly retrospective, that have a limited ability for causal inference. SCHEER acknowledged that it is currently impossible to produce level of evidence I/II prospective studies, because BIA-ALCL is an uncommon disease and the highest risk macrotextured devices are either recalled or represent a miniscule portion of current market share. Level III studies are difficult to produce because data collection is limited by the lack of implant registries and of transparent access to data by government authorities. Hence, level IV/V studies are the most readily available for extrapolation of data. Randomized control trials of textured surface implants on humans may now be considered unethical and unachievable since highest-risk devices have been withdrawn globally, concluding that the *moderate level* is likely the strongest possible evidence achievable [15]. SCHEER has not recommended a full textured implant recall nor prophylactic explantation because it is not in their mandate, but deferred this to National Regulatory Authorities.

In order to conclude justification for removal of textured surface implants, it is important to review precedence for authority guidance on prophylactic explantation. In 2010, the Agence Nationale de Sécurité du Médicament et des produits de Santé (ANSM) recalled from the market and recommended explantation of Poly Implant Prothèse BIs because of the high risk of premature rupture (approaching 50% at one year) and the use of low-quality industrial-grade silicone gel. The *Treaty on the functioning of the European Union*, Art. 191, states that the “precautionary principle” can be applied “when a product may have a dangerous effect, identified by a scientific and objective evaluation, if this evaluation does not allow the risk to be determined with sufficient certainty” [16].

In 2015, the Dutch National Institute for Public Health and the Environment asked the Independent Clinical Expert Advisory Group (ICEAG), to assess the carcinogenic risks of man-made mineral fibers (MMMFs) found on Silimed BIs surface [17]. According to the World Health Organization, “acceptable cancer risk” for lifetime exposure in the general population is 1 in 1,000,000 [18]. The RIVM toxicologic analysis found MMMFs on textured SBIs, but also on polyurethane-coated which were not detected by TÜV, and interestingly both authorities did not find these fibers on smooth surface implants. It was concluded that total maximum exposure with two Silimed implants was up to (73 glass fibers + 1,440 rockwool fibers [the most potent type] × 2 =) 3026 fibers, with a cancer risk of 9 per 1,000,000 (0.0009%) which is greater than the “acceptable cancer risk”. Assuming that smaller rockwool fibers are not MMMFs (*i.e.,* non-carcinogenic), reduces exposure to 146 large glass mineral fibers (≥ 20 μm) resulting in a cancer risk of 4.42 per 10,000,000 (or 0.000044%), which is lower than the acceptable cancer risk. The authors of this study noted that these estimates are subject to many uncertainties and limitations like the intraperitoneal introduction of fibers in rat models, rather than the breast/muscle position in a human model. MMMFs found on Silimed implants, because of their large size, may lead to higher toxicity through increased biopersistence or to lower toxicity if encapsulated [17]. The ICEAG concluded that since the risk is “very small and around the acceptability limit” decisions about risk management should be jointly made by patients and their treating physicians. Although a recent study clarified that the carcinogenic risk in patients bearing the investigated type of polyurethane-coated Silimed implants is much higher than presumed, reaching up to 1 in 2,382 [19], regulatory authorities did not recommend precautionary removal of BIs at risk of BIA-ALCL. Despite that, to prevent BIA-ALCL, the ANSM banned the use of macrotextured implants in April 2019, and the US FDA requested a class I recall of Allergan textured devices in July 2019, but none recommended a precautionary removal of BIs considered at high risk due to a lack of data on the risk and benefits.

Implant removal/exchange already occurs in woman bearing BIs during their life, independently of BIA-ALCL risk, most notably for capsular contracture, implant rupture, implant malposition, and patient preference for size adjustment. Our literature review highlighted a mean age for women undergoing first BI placement (A1P) between 38 (USA) and 40 years (Italy), and a modern BIs lifespan (BIL) of 9.3 years, which combined to a woman mean life expectancy (WLE) of 85 years, suggests that many women with BIs will undergo to 4.9–5 times replacement in their lifetimes if the BIs are not removed [11].

Several limitations are noted, as these estimates on A1P were inferred from best available data due to a lack of publicly available information from breast implant manufacturers. Industry-sponsored core studies in literature report varying implant rupture, explantation, exchange, and reoperation rates and are divided into primary/revision augmentations and primary/revision reconstructions. All of these rates are reported to significantly rise by 10 years after implantation (Table 4).

**Table 4:** Comparison of breast implant rupture rates, explantation rates (with or without implant exchange, explantation only and implant exchange only) and reoperation rates across primary augmentation, revision augmentation, primary reconstruction and revision reconstruction patients.

	Tested implant	Manufacturer	Implant shape	Primary Augmentation	Revision Augmentation	Primary Reconstruction	Revision Reconstruction	Follow-up
*Breast implant rupture rates*								
Cunningham et al. [20]	MemoryGel	Mentor	Round	0.5%	7.7%	0.9%	0%	3 years
Cunningham et al. [21]	MemoryGel	Mentor	Round	1.1%	11.6%	3.8%	5.9%	6 years
Caplin [22]	MemoryGel	Mentor	Round	10%	–	15%	–	8 years
Caplin et al. [14]	MemoryGel	Mentor	Round	24.2%	23.7%	32.7%	38.7%	10 years
Hammond et al. [23]	MemoryShape (Contour Profile Gel)	Mentor	Shaped	2.1%	2.9%	1.5%	0%	6 years
Caplin [22]	MemoryShape (Contour Profile Gel)	Mentor	Shaped	3%	–	10%	–	8 years
Hammond et al. [24]	MemoryShape (Contour Profile Gel)	Mentor	Shaped	6.6%	9.6%	18.9%	0%	10 years
Quirós et al. [25]	Motiva SmoothSilk/ SilkSurface	Establishment Labs	Round	0%	–	–	–	6 years
Nichter et al. [26]	Structured Breast Implant	IDEAL IMPLANT	Round	1.8%	4.7%	–	–	6 years
Han et al. [27]	BellaGel	Hans Biomed	Round and Shaped	0%	–	0%	–	4 years
Oh et al. [28]	BellaGel	Hans Biomed	Round and Shaped	0%	–	3.77%	–	6 years
El-Haddad et al. [29]	Sebbin Silicone Textured and Smooth	Sebbin	Round	7.4%		21.2%		10 years
Duteille et al. [30]	Cristalline Paragel	Eurosilicone S.A.S. (GC Aesthetics)	Round and Anatomical	0.8%	0%	0%	0%	5 years
Duteille et al. [31]	Cristalline Paragel	Eurosilicone S.A.S. (GC Aesthetics)	Round and Anatomical	1.9%	0%	0%	0%	8 years
Duteille et al. [32]	Cristalline Paragel	Eurosilicone S.A.S. (GC Aesthetics)	Round and Anatomical	4.9%	2.3%	0%	0%	10 years
Spear et al. [33]	Inamed Silicone Textured and Smooth	Inamed (Allergan)	Round and Shaped	5.5%	2.3%	9.3%		6 years
Spear et al. [34]	Natrelle Round	Allergan	Round	9.3%	5.4%	35.4%		10 years
Bengtson et al. [35]	Inamed Style 410	Inamed (Allergan)	Anatomical	0.7%	2.2%	1.3%	0%	3 years
Maxwell et al. [36]	Natrelle Style 410	Allergan	Anatomical	5.0%	5.0%	7.5%	14.3%	6 years
Maxwell et al. [37]	Natrelle Style 410	Allergan	Anatomical	17.7%	14.7%	12.4%	19.6%	10 years
Stevens et al. [38]	Sientra/Silimed	Sientra	Round and Shaped	2.0%	1.5%	1.4%	0%	5 years
Stevens et al. [39]	High-Strength Cohesive (HSC), High-Strength Cohesive Plus (HSC+)	Sientra	Round and Shaped	4.3%	3.3%	1.2%	14.5%	8 years
Stevens et al. [39]	High-Strength Cohesive (HSC), High-Strength Cohesive Plus (HSC+)	Sientra	Round and Shaped	7.8%	5.2%	9.8%	–	10 years
*Explantation rates (with or without implant exchange)*								
Hammond et al. [23]	MemoryShape (Contour Profile Gel)	Mentor	Shaped	7.0%	13.6%	21.8%	34.2%	6 years
Hammond et al. [24]	MemoryShape (Contour Profile Gel)	Mentor	Shaped	9.2%	25.9%	34.1%	49.0%	10 years
Caplin [22]	MemoryGel	Mentor	Round	10.3%	22.1%	30.4%	35.7%	9 years
Caplin [22]	MemoryShape (Contour Profile Gel)	Mentor	Shaped	8.6%	23.1%	34.9%	50.8%	9 years
Short et al. [40]	MemoryGel	Mentor	Round	4.2%	7.7%	12.8%	16.6%	10 years
Caplin et al. [14]	MemoryGel	Mentor	Round	11.6%	24.1%	33.4%	37.8%	10 years
Han et al. [27]	BellaGel	Hans Biomed	Round and Shaped	6.5%	–	16.7%	–	4 years
El-Haddad et al. [29]	Sebbin Silicone Textured and Smooth	Sebbin	Round	21.3%		64.1%		10 years
Duteille et al. [30]	Cristalline Paragel	Eurosilicone S.A.S. (GC Aesthetics)	Round and Anatomical	8.8%	22.9%	14.7%	15.2%	5 years
Duteille et al. [31]	Cristalline Paragel	Eurosilicone S.A.S. (GC Aesthetics)	Round and Anatomical	6.0%	–	13.8%	–	8 years
Duteille et al. [32]	Cristalline Paragel	Eurosilicone S.A.S. (GC Aesthetics)	Round and Anatomical	11.9%	–	–	–	10 years
Stevens et al. [38]	Sientra/Silimed	Sientra	Round and Shaped	8.7%	17.3%	31.0%	38.6%	5 years
Stevens et al. [39]	High-Strength Cohesive (HSC), High-Strength Cohesive Plus (HSC+)	Sientra	Round and Shaped	13.9%	23.8%	36.4%	46.9%	9 years
Stevens et al. [41]	High-Strength Cohesive (HSC), High-Strength Cohesive Plus (HSC+)	Sientra	Round and Shaped	16.0%	26.0%	36.5%	50.6%	10 years
*Explantation only rate*s								
Cunningham et al. [20]	MemoryGel	Mentor	Round	4.9%	13.4%	12.7%	13.7%	3 years
Cunningham et al. [21]	MemoryGel	Mentor	Round	4.0%	8.1%	8.0%	7.2%	6 years
Short et al. [40]	MemoryGel	Mentor	Round	4.2%	7.7%	12.8%	16.6%	10 years
Han et al. [27]	BellaGel	Hans Biomed	Round and Shaped	0%	–	5.6%	–	4 years
Duteille et al. [31]	Cristalline Paragel	Eurosilicone S.A.S. (GC Aesthetics)	Round and Anatomical	8.2%	–	20.4%	–	8 years
Bondurant et al. [42]	McGhan AR90	Inamed (Allergan)	Round and Shaped	6.0%		14.0%		5 years
Spear et al. [33]	Inamed Silicone Textured and Smooth	Inamed (Allergan)	Round and Shaped	2.8%	4.4%	7.7%		6 years
Bengtson et al. [35]	Inamed Style 410	Inamed (Allergan)	Anatomical	0.7%	2.7%	3.5%	0%	3 years
Maxwell et al. [36]	Natrelle Style 410	Allergan	Anatomical	0.7%	3.6%	4.6%	1.9%	6 years
Maxwell et al. [37]	Natrelle Style 410	Allergan	Anatomical	16.8%	27.8%	34.3%	39.3%	10 years
*Implant exchange only rates*								
Cunningham et al. [20]	MemoryGel	Mentor	Round	2.8%	6.5%	7.4%	8.8%	3 years
Cunningham et al. [21]	MemoryGel	Mentor	Round	3.9%	10.0%	10.4%	16.7%	6 years
Han et al. [27]	BellaGel	Hans Biomed	Round and Shaped	6.5%	–	11.1%	–	4 years
Spear et al. [33]	Inamed Silicone Textured and Smooth	Inamed (Allergan)	Round and Shaped	10.0%	18.6%	22.3%		6 years
Bengtson et al. [35]	Inamed Style 410	Inamed (Allergan)	Anatomical	4.7%	8.3%	13.8%	15.4%	3 years
Maxwell et al. [36]	Natrelle Style 410	Allergan	Anatomical	9.6%	20.2%	23.7%	20.1%	6 years
Maxwell et al. [37]	Natrelle Style 410	Allergan	Anatomical	16.8%	27.8%	34.3%	39.3%	10 years
*Reoperation rates *								
Cunningham et al. [20]	MemoryGel	Mentor	Round	15.4%	28.0%	27.0%	29.1%	3 years
Cunningham et al. [21]	MemoryGel	Mentor	Round	19.4%	34.2%	33.9%	36.2%	6 years
Caplin [22]	MemoryGel	Mentor	Round	21.2%	41.6%	45.5%	50.7%	9 years
Short et al. [40]	MemoryGel	Mentor	Round	10.5%	14.1%	20.8%	25.0%	10 years
Caplin et al. [14]	MemoryGel	Mentor	Round	25.5%	43.6%	49.0%	50.7%	10 years
Hammond et al. [23]	MemoryShape (Contour Profile Gel)	Mentor	Shaped	18.1%	24.1%	44.5%	45.4%	6 years
Caplin [22]	MemoryShape (Contour Profile Gel)	Mentor	Shaped	23.4%	33.0%	51.6%	61.6%	9 years
Hammond et al. [24]	MemoryShape (Contour Profile Gel)	Mentor	Shaped	22.3%	35.0%	52.7%	59.7%	10 years
Quirós et al. [25]	Motiva SmoothSilk/ SilkSurface	Establishment Labs	Round	9.4%	–	–	–	6 years
Han et al. [27]	BellaGel	Hans Biomed	Round and Shaped	6.5%	–	16.7%	–	4 years
Duteille et al. [30]	Cristalline Paragel	Eurosilicone S.A.S. (GC Aesthetics)	Round and Anatomical	1.9%	17.6%	18.0%	–	5 years
Duteille et al. [31]	Cristalline Paragel	Eurosilicone S.A.S. (GC Aesthetics)	Round and Anatomical	8.2%	20.4%	24.1%	25.9%	8 years
Duteille et al. [32]	Cristalline Paragel	Eurosilicone S.A.S. (GC Aesthetics)	Round and Anatomical	13.3%	23.8%	31.6%	38.4%	10 years
Nichter et al. [26]	Structured Breast Implant	IDEAL IMPLANT	Round	27.6%	39.3%	–	–	6 years
Stevens et al. [38]	Sientra/Silimed	Sientra	Round and Shaped	16.6%	29.7%	42.7%	47.8%	5 years
Stevens et al. [39]	High-Strength Cohesive (HSC), High-Strength Cohesive Plus (HSC+)	Sientra	Round and Shaped	22.2%	36.6%	47.9%	57.4%	9 years
Stevens et al. [41]	High-Strength Cohesive (HSC), High-Strength Cohesive Plus (HSC+)	Sientra	Round and Shaped	24.0%	38.8%	48.2%	56.7%	10 years
Spear et al. [33]	Inamed Silicone Textured and Smooth	Inamed (Allergan)	Round and Shaped	28.0%	40.3%	51.9%		6 years
Spear et al. [34]	Natrelle	Allergan	Round	36.1%	46.0%	71.5%		10 years
Bengtson et al. [35]	Inamed Style 410	Inamed (Allergan)	Anatomical	12.5%	21.1%	31.8%	19.8%	3 years
Maxwell et al. [36]	Natrelle Style 410	Allergan	Anatomical	19.4%	35.1%	43.1%	33.7%	6 years
Maxwell et al. [37]	Natrelle Style 410	Allergan	Anatomical	29.7%	47.3%	54.6%	48.5%	10 years

“Data were retrieved from core studies and post-approval studies. “–“ is used when data in not available. The terms “Anatomical” and “Shaped” are used interchangeably throughout the table.

There are several arguments against prophylactic BI removal. Wixtrom et al. [7] found that cosmetic breast surgery was associated with a mortality rate of 1:72,000 procedures (0.0014%), though the author failed to include the percentage of BI procedures out of the overall number of cosmetic breast surgeries in the estimation. Frail and comorbid patients are at greater risk of death with a plastic surgery procedure as with any type of surgery, and therefore, patient selection is critical when weighing risks and benefits of any procedure. Perioperative and anesthesia-related mortality was recently estimated at 1:250,000 patients without systemic disease [43]. With BIA-ALCL mortality rate of 3% and a chemotherapy/radiotherapy rate of 15%, our findings of 0% BISM rate in 99,690 surgeries suggest that explantation/replacement is likely a safe option for patients at risk of BIA-ALCL, similar to what is also being performed for patients with capsule contracture or in other cases requiring implant revisional surgery [1]. It should be duly noted how any revisional surgery can be associated with surgical risks, such as pneumothorax 0.03-04% and infections 1.9-2.5% [44]. These risks, together with psychosocial and aesthetic discomfort, are important considerations for women deciding on prophylactic explantation with or without replacement. Long-term outcomes of risk mitigation strategies were not intended to be investigated in this study, but deserve to be analyzed in future studies and discussed with patients during consultation.

Some authors have argued that there is a clear rationale against prophylactic explantation. Most patients, even with high-risk textured devices, will not develop BIA-ALCL [45]. Estimated risk varies from 1:86,029 for Mentor Siltex implants and 1:2,832 for polyurethane Silimed implants [19]. Cordeiro et al. [3] reported a risk of 1 in 350 in a cohort of 3,546 breast reconstruction patients with Allergan macrotextured Biocell devices. This last sub-cohort demonstrates the highest risk per device to date, as the US FDA estimates that approximately 91% of known BIA-ALCL cases involved a history of Biocell implantation when the clinical history was known. Nevertheless, because of missing sales data and the lack of implant registries, it is not possible to clearly define surface-specific incidence rates. Therefore, “high-risk” implants can be considered either only those specific branded types associated with most BIA-ALCL cases, such as Allergan BIOCELL or Silimed Polyurethane-coated devices, or all the textured devices with high surface roughness, so-called macrotextured, hence including more than the previous two [46].

It is important to consider the potential effect of genetic predisposition of cancer patients, including *TP53 and BRCA1/2* mutations, connected to other types of cancer, which have found to be associated with BIA-ALCL [47]. Consequently, “high-risk” patients are to be considered those not only bearing one of such textured implants at high risk but also showing other risk factors as cancer-predisposing mutations. Those factors along with patient’s age and comorbidities should all be considered and taken into account when deciding on prophylactic explantation.

Long-term data on risk mitigation strategies are still lacking. BIA-ALCL has developed after explantation without capsulectomy [48], after incomplete capsulectomy [49], and after simultaneous implant replacement from textured to smooth, showing limited evidence about the specifics of what is the optimal total capsulectomy to be performed [50]. In patients at high risk for breast cancer, such as *BRCA1/2* mutations, prophylactic mastectomy is indicated, heavily reducing though not completely eradicating the risk of cancer. It is therefore reasonable to consider for patients at high risk for BIA-ALCL, a prophylactic total capsulectomy with explant/exchange of a prosthesis, which carries a much lower risk compared to a bilateral mastectomy and oophorectomy. High level evidence to assess prophylactic explantation and risk reduction remains impossible to obtain, as it would require prospective clinical trials with long-term follow-ups using now-banned devices. Although our study is not able to guarantee a causal relationship between explantation/total capsulectomy and risk reduction, due to its non-prospective nature, this does not mean that the acquired data should not be considered when the patient and the physician are taking a decision. Based upon the evidence that women with BIs undergo several revisional surgeries or implant exchanges, given the neglectable BISM risk, it is reasonable to consider, as part of the informed consent, a prophylactic indication for exchanging textured implants in high-risk patients. A total capsulectomy either as *complete intact,* can be considered *reasonable and appropriate*, based upon a patients comorbidities and surgical risk stratification. Importantly, *en bloc* capsulectomy involving margin evaluation and oncologic technique is reserved for management of confirmed BIA-ALCL cases [51].

Finally, it is beyond the scope of this manuscript to evaluate the financial and economic impact of wide adoption of prophylactic explantation; however, it cannot be dismissed. In the USA, textured BI usage peaked at 22.89% of all placements in 2016, dwindling thereafter, reaching 3.61% in 2019 [52]. Due to the mean age of presentation of BIA-ALCL, thousands of cases are still predicted to manifest over the next decade. According to the American Society for Aesthetic Plastic Surgery, with 66,982 explantation in 2019, it was the 8th most performed surgical procedure by plastic surgeons [53]. Wider adoption of prophylactic management of high-risk devices may have a measurable impact on the American healthcare system. For Europe, where textured devices represent the vast majority of all BIs, it might quickly outstrip the capacity of healthcare systems.

## Conclusions

Primary and revisional BI surgeries are to be considered safe, and prophylactic removal of high-risk BIs should not be discounted on the basis of the potential mortality risks alone. Some of the problems that remain to be solved include the coverage of the costs, detrimental resource effect to the healthcare system, and the development of an accurate list of all high-risk implants. Questions remain as to whether a specific type or extent of capsule resection is required for risk reduction of existing macrotextured implants as limited case reports exist of disease manifestation years following total capsulectomy and no standardized approach to capsulectomy is recognized internationally. Data are lacking whether explantation provides a BIA-ALCL risk reduction benefit, though clinical trial is not feasible given the low prevalence and significant follow-up required to assess any outcome. While contralateral prophylactic capsulectomy is indicated in known BIA-ALCL disease, we recommend that asymptomatic patients with the existing macrotextured implants do have a relative indication for explantation and total capsulectomy. The final decision should be shared between patient and surgeon following an evaluation of the surgical risks, comorbidities, and benefits based upon a patient’s goals.
